# Brachial Artery Constriction during Brachial Artery Reactivity Testing Predicts Major Adverse Clinical Outcomes in Women with Suspected Myocardial Ischemia: Results from the NHLBI-Sponsored Women’s Ischemia Syndrome Evaluation (WISE) Study

**DOI:** 10.1371/journal.pone.0074585

**Published:** 2013-09-18

**Authors:** Tara L. Sedlak, B. Delia Johnson, Carl J. Pepine, Steven E. Reis, C. Noel Bairey Merz

**Affiliations:** 1 Department of Medicine, Vancouver General Hospital, Vancouver, British Columbia, Canada; 2 Department of Epidemiology, University of Pittsburgh, Pittsburgh, Pennsylvania, United States of America; 3 Department of Medicine, University of Florida, Gainesville, Florida, United States of America; 4 Department of Medicine, Cedars-Sinai Heart Institute, Los Angeles, California, United States of America; University of Tor Vergata, Italy

## Abstract

**Background:**

Limited brachial artery (BA) flow-mediated dilation during brachial artery reactivity testing (BART) has been linked to increased cardiovascular risk. We report on the phenomenon of BA constriction (BAC) following hyperemia.

**Objectives:**

To determine whether BAC predicts adverse CV outcomes and/or mortality in the women’s ischemic Syndrome Evaluation Study (WISE). Further, as a secondary objective we sought to determine the risk factors associated with BAC.

**Methods:**

We performed BART on 377 women with chest pain referred for coronary angiography and followed for a median of 9.5 years. Forearm ischemia was induced with 4 minutes occlusion by a cuff placed distal to the BA and inflated to 40mm Hg > systolic pressure. BAC was defined as >4.8% artery constriction following release of the cuff. The main outcome was major adverse events (MACE) including all-cause mortality, non-fatal MI, non-fatal stroke, or hospitalization for heart failure.

**Results:**

BA diameter change ranged from -20.6% to +44.9%, and 41 (11%) women experienced BAC. Obstructive CAD and traditional CAD risk factors were not predictive of BAC. Overall, 39% of women with BAC experienced MACE vs. 22% without BAC (p=0.004). In multivariate Cox proportional hazards regression, BAC was a significant independent predictor of MACE (p=0.018) when adjusting for obstructive CAD and traditional risk factors.

**Conclusions:**

BAC predicts almost double the risk for major adverse events compared to patients without BAC. This risk was not accounted for by CAD or traditional risk factors. The novel risk marker of BAC requires further investigation in women.

## Introduction

Brachial artery reactivity testing (BART) for measurement of flow-mediated dilation (FMD) has been widely used in clinical research as a non-invasive measure of endothelial function. A typical FMD response in persons with a healthy endothelium is at least a 5% to 10% increase in the brachial artery diameter following release of a constrictive cuff (reactive hyperemia). Inadequate dilation following hyperemia signals endothelial dysfunction and has been linked to cardiovascular (CV) risk factors and conditions associated with atherosclerosis; inadequate peripheral FMD response has also been associated with coronary endothelial dysfunction [1-3]. We previously reported on resting brachial artery diameter and FMD in 377 women with chest pain from the Women’s Ischemic Syndrome Evaluation Study (WISE) who underwent coronary angiography and risk factor assessment [4]. While impaired FMD was weakly associated with obstructive CAD, after adjustment for resting brachial artery diameter, FMD was not an independent predictor of obstructive CAD in women with chest pain.

Although it is generally assumed that release of the blood pressure cuff should result in an increase in the brachial artery diameter, constriction has been encountered in prior studies. This phenomenon has been largely ignored [3,5,6] or attributed to blunted endothelial function [7,8]. A blunted vasodilator response following cuff release has been shown to correlate with cardiovascular events [1-3]. The present study presents novel data showing a comparatively high rate of brachial artery constriction in a unique population of women with ischemia undergoing coronary angiography.

The WISE study is a multi-center study that aims to improve the diagnostic reliability of cardiovascular testing in the evaluation of ischemic heart disease in women. Using standard procedures available in 1996-1999, brachial reactivity testing (BART) was performed at baseline in a subsample of the study population. The purpose of the present paper is to investigate the phenomenon of Brachial Artery Constriction (BAC) in the WISE and to ask the following questions: (1) What are the risk factors associated with BAC, and (2) Does BAC predict adverse CV outcomes and/or mortality?

## Methods

### Study Population

The study population consisted of 377 participants in the WISE study who received BART at baseline evaluation. WISE is a National Heart, Lung, and Blood Institute –sponsored multi-center study of 936 women undergoing clinically ordered coronary angiography for suspected myocardial ischemia [9]. The study population was a convenience sample based on interest and availability of equipment within each clinical site. The four clinical enrollment sites in addition to inclusion and exclusion criteria have been described previously [9-11]. All women provided signed informed consent for baseline evaluations and follow-up by using forms and procedures in accordance with institutional guidelines and approved by the institutional review board at each WISE clinical site including University of Pittsburgh Medical Center, the Allegheny County General Hospital (both located in Pittsburgh, PA), the University of Florida, Gainesville, FL, and the University of Alabama at Birmingham, AL. The baseline demographic, risk factor, and quantitative assessment of coronary angiography measurements have also been described previously [9-11].

### Brachial Artery Reactivity Testing (BART)

All vasoactive medications were withheld for ≥24 hours if short-acting and ≥48 hours if long-acting. Resting brachial artery diameter was measured with B-mode ultrasonography with a 7.5-MHz probe with imaging recorded on Super VHS tapes. Blood pressure cuff was placed distal to the brachial artery and inflated to 44 mm Hg greater than systolic blood pressure for 4 minutes. We acknowledge that other studies use a 5 minute occlusion time [7,8], therefore our extent of reactive hyperemia may be less and our results may not be directly comparable to prior studies. Brachial artery diameter was reassessed for 2 minutes after cuff deflation. Offline quantitative analysis was performed at a core laboratory (University of Pittsburgh) by investigators masked to subject identifiers. Images were digitized, and calibrated electronic calipers were used to measure brachial artery diameter with the intima-media interface to define arterial borders. Three measurements were made in each analyzed frame and averaged (intraclass correlation coefficient between 2 independent observers = 0.94). We have previously published on our core lab reproducibility which was obtained using repeated reading of the same image not repeat testing [4]. FMD was calculated as FMD = 100x (peak diameter after cuff deflation – resting diameter) / resting diameter. FMD was treated as a continuous variable for our analysis. We defined BAC as > 4.8% constriction following release of the blood pressure cuff. The reason for choosing this cut-point was that it represented 1 standard deviation below the mean, sufficient to distinguish it from possible measurement error or other sources of random variation. This cut-point was further verified by ROC analysis to give the best prediction of MACE. While we have called this BAC for convenience, we realize that we cannot confirm with certainty that BAC represents true constriction versus failure to dilate following release of the blood pressure cuff.

### Follow-up procedures

Follow-up was conducted by experienced site nurses or physicians through telephone and/or mail contact at 6 weeks and then yearly thereafter. Follow-up consisted of a scripted interview. Each woman was queried about symptoms, medication use, CV events since last contact, hospitalizations, and diagnostic or revascularization procedures. In the event of death, a death certificate was obtained. During this period, follow-up information was collected for a median of 6.8 years. Subsequently, we conducted a National Death Index (NDI) search for all women who were still alive and obtained death certificates. This extended the follow-up, only for mortality, to a median of 9.55 years (interquartile range 8.2-10.3 years). WISE investigators blinded to identifying and diagnostic information classified all deaths as CV versus non-CV. A major event was defined as a death, non-fatal MI, non-fatal stroke, or hospitalization for heart failure. A CV event was defined as CV-related mortality, non-fatal MI, non-fatal stroke, or hospitalization for heart failure.

### Statistical analysis

We used ROC analysis to estimate the optimal cut-point for defining BAC as a predictor of MACE. The resulting cut-point of >4.8% constriction yielded 41 women (11%) with BAC. Because of the small sample size of the BAC group, the area under the curve (AUC) is small but significantly different from chance (0.55 [95% confidence interval=0.51, 0.59]).

Comparisons between women with and without BAC were performed by the t-test for continuous measures and by chi square for dichotomous measures. Because age is a major correlate in WISE of demographic characteristics and CAD risk factors, all p-values in Table 1 (with the exception of age and postmenopausal status) were adjusted for age using logistic regression. A multivariable model predicting the presence or absence of BAC based on major risk factors for CAD was developed using logistic regression. In addition, we also examined the correlation between nitroglycerin mediated dilation and outcomes as well as baseline diameter and events.

**Table 1:** Baseline Characteristics by Brachial Artery Vasoconstriction (BAC).

**Characteristic**	**No BAC (n=336**)	**BAC (n=41**)	**Age-Adjusted p-Value**
Age	57 ± 11	60 ± 13	0.16
Postmenopausal (%)	72	85	0.08
Non-white race^a^ (%)	13	10	0.64
Obstructive CAD^b^ (%)	36	36	0.65
Diabetes (%)	18	22	0.63
≥3 CAD risk factors^c^ (%)	63	49	0.09
Hx^d^ hypertension (%)	54	56	0.98
Hx^d^ dyslipidemia (%)	50	38	0.07
Family hx of CAD (%)	72	54	0.017
BMI^e^ ≥ 30 (%)	38	34	0.67
Ever smoked (%)	55	58	0.62
Prior MI^f^ or revasc (%)	26	20	0.33
Recent aspirin use (%)	51	68	0.06
Recent psych med use (%)	19	32	0.046
Insulin levels uIU/mL	10 ± 10	16 ± 14	0.031
Hs-CRP mg/L	7 ± 13	11 ± 16	0.18
Pulmonary disease (%)	4	15	0.015
Estradiol levels pg/mL	42 ± 51	19 ± 15	0.007
FSH^g^ levels mIU/mL	32 ± 24	47 ± 30	0.002
Postmeno HT^h^ use (%)	48	34	0.19

^a^ Non-white race includes 45 African American and 4 Hispanic women; ^b^ Obstructive CAD defined as ≥50% stenosis in ≥1 major epicardial vessel; ^c^ CAD risk factors includes diabetes, hx of dyslipidemia, hx of hypertension, family hx of CAD, BMI ≥30, ever smoking, and prior MI or revascularization; ^d^ Hx=history; ^e^ BMI=body mass index; ^f^ MI=myocardial infarction; ^g^ FSH=follicle stimulating hormone; ^h^ HT=hormone therapy.

Event rates were estimated using the Kaplan-Meier method and the log-rank statistic to compare event-free survival time in women with and without BAC. We used Cox proportional hazards regression to assess the relationship between FMD or BAC and major cardiovascular outcomes unadjusted as well as adjusted for age. We also developed multivariable Cox proportional hazards models based on the major risk factors for major CV events (using Framingham risk criteria) in addition to BAC. When a major risk factor was not significant, we used proxy variables, for example, recent use of anti-hypertension medication instead of history of hypertension and recent statin use instead of history of dyslipidemia. We then forced the CAD severity score (log transformation) into the model (Table 2, Model 2). In addition, we evaluated other log transformations and interaction terms. The validity of the proportional hazards assumption of invariant relative risk was tested and found to be satisfactory. All p-values of <0.05 were considered statistically significant. All analyses were performed using the SAS 9.2 software (SAS, Cary, NC).

**Table 2:** Independent Predictors of Major Cardiovascular Events: Two Models.

**Predictors**	Model 1		Model 2	
	HR (95% CI)	p-Value	HR (95% CI)	p-Value
BAC^a^	2.21 (1.30, 3.78)	0.004	1.96 (1.12, 3.43)	0.018
Age	1.03 (1.01, 1.05)	0.002	1.02 (1.00, 1.04)	0.058
Diabetes	1.59 (0.99, 2.53)	0.052	1.41 (0.87, 2.30)	0.16
Ever smoked	1.62 (1.05, 2.49)	0.029	1.43 (0.91, 2.23)	0.12
Recent anti-HTN^b^ meds	1.89 (1.16, 3.08)	0.011	1.70 (1.03, 2.82)	0.039
Recent statin use	1.79 (1.14, 2.81)	0.011	1.31 (0.80, 2.14)	0.28
CAD^c^ severity score (log)	-	-	1.85 (1.40, 2.45)	<0.001

^a^ BAC = Brachial artery constriction; ^b^ HTN = hypertension; ^c^ CAD = coronary artery disease

## Results

### Characteristics

The mean age was 58±11, ranging from 21 to 83 years. Most women (74%) were postmenopausal, 13% were non-white, primarily African American, and 59% had 3 or more CAD risk factors that included diabetes, dyslipidemia, hypertension, family history of CAD, BMI ≥ 30, ever smoking, and prior MI or revascularization. A total of 137 (36%) had obstructive CAD defined as ≥50% stenosis in ≥1 epicardial vessel. The mean ejection fraction was 65.2% ± 9.8%.

**Figure 1:**
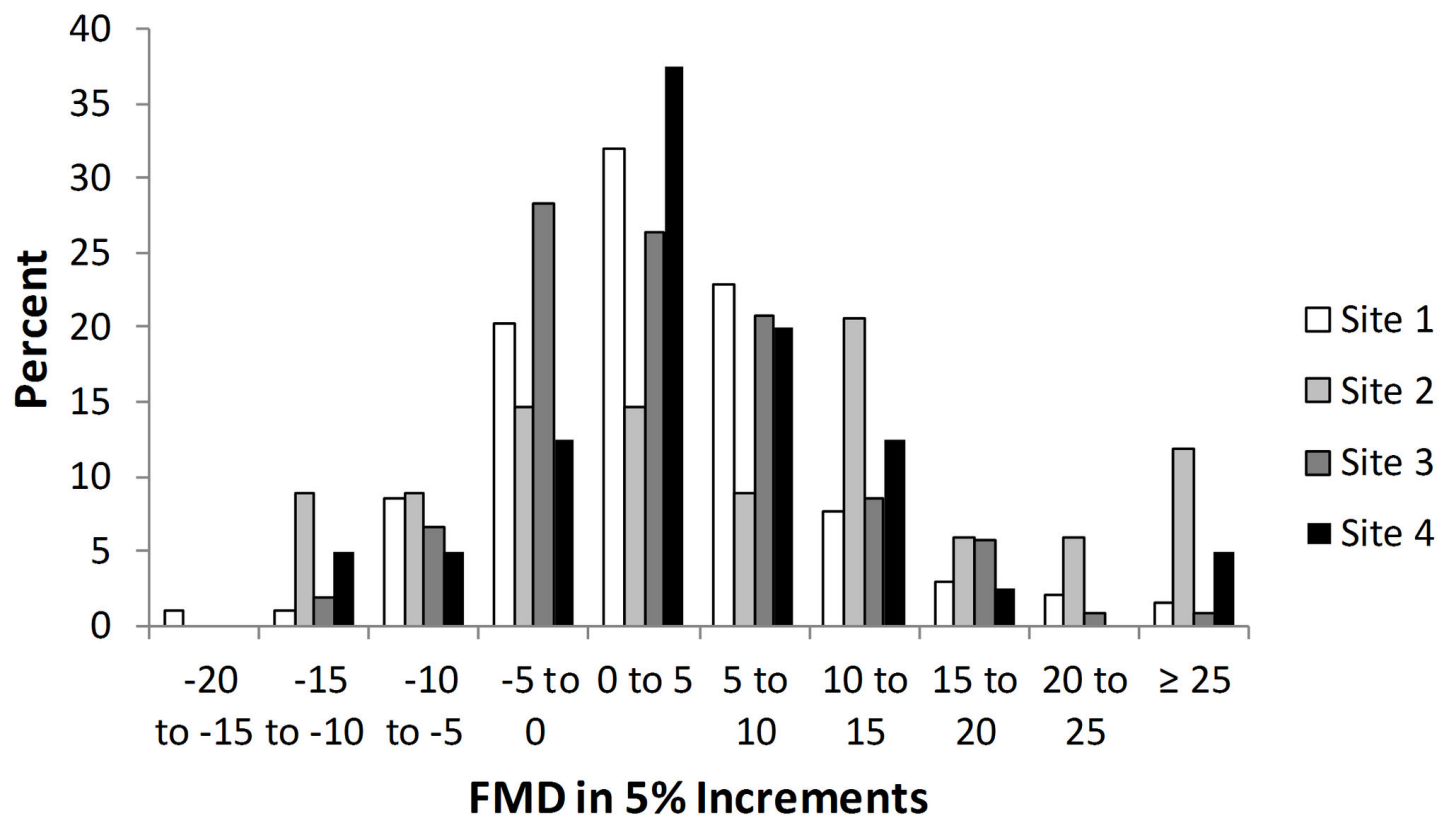
Distribution of Brachial Artery Reactivity by Clinical Site.

The mean FMD was 3.8 ± 8.7, with a median of 2.8 and interquartile range of -1.3 to 7.9. Of the 377 women, 231 (61%) had FMD<5%, while 41 (11%) had BAC. There was no difference in FMD distribution or presence of BAC across clinical sites (p=0.50) (Figure 1).

Compared to the 559 WISE women not undergoing BART, the 377 with BART testing were more likely to be white (87% vs. 77%, p=0.0002). Although not differing in age or presence of obstructive CAD, the women undergoing BART had, on the average, more positive health indicators: they had a lower CAD severity score (13.3±13.3 vs. 15.9±15.5, p=0.007), lower systolic blood pressure (134±20 vs. 139±22, p=0.0007), better functional capacity (Duke Activity Score Index (DASI) of 21.9±14.8 vs. 18.5±14.5, p=0.0006), and were less likely to have diabetes (18% vs. 29%, p=0.0001) or take psychoactive medications (20% vs. 37%, p<0.0001), anti-hypertension medications (60% vs. 72%, p=0.0002), nitrates (26% vs. 41%, p<0.0001), or aspirin (53% vs. 65%, p=0.0001). These differences remained consistent even after statistical adjustment for ethnic origin. Much of these differences can be explained by the relatively low number of BARTs performed at the Alabama WISE clinical site, a Southern state with a large African American population, where BART was done on only 14% of its WISE women, compared to Allegheny General Hospital in Pittsburgh that performed BART on 85% of its WISE population, with the other clinical sites at intermediate percentages.

### Predictors of BAC

Table 1 gives the baseline characteristics for women with versus without BAC. Although the women with BAC were slightly older and more likely to be postmenopausal, these differences were not statistically significant. Because of these slight differences, however, we statistically adjusted all p-values for age. No traditional or non-traditional CAD risk factors aside from those in Table 1 with a p<0.05 were significantly associated with BAC, including chronic use of vasoactive medications such as statins, ACE inhibitors, beta blockers, or angiotensin receptor blockers. In multivariate analysis, only insulin levels (odds ratio = 1.05 [95% confidence interval = 1.01, 1.08], p=0.010), pulmonary disease (5.07 [1.35, 19.050], p=0.016), and family history of CAD (0.38 [0.14, 1.00], p=0.050) were independent predictors of BAC. The model explained 13% of the variance and the c-statistic was 0.70 (data not shown).

### Prediction of Major Adverse Events

Over a median of 9.5 years, a total of 83 (22%) women had a major adverse event, including 57 (15%) deaths, and 26 (7%) had a non-fatal MI, stroke, or hospitalization for heart failure. The number of women with non-fatal events is an underestimate since these data were only collected for a median of 6.8 years of telephone and/or mail follow-up compared to the longer-term death follow-up.

In unadjusted analyses, lower FMD was predictive of CV-related death (HR = 0.95 [0.92, 0.99], p = 0.025). However, after adjusting for age, this result became non-significant. Conversely, FMD did not predict major adverse events (HR = 0.98 [95% confidence interval = 0.96, 1.01], p = 0.16), cardiovascular events (HR = 0.97 [0.94, 1.005], p = 0.10), or all-cause mortality (HR = 0.98 [0.95, 1.01], p = 0.13) (Data not shown).

**Figure 2:**
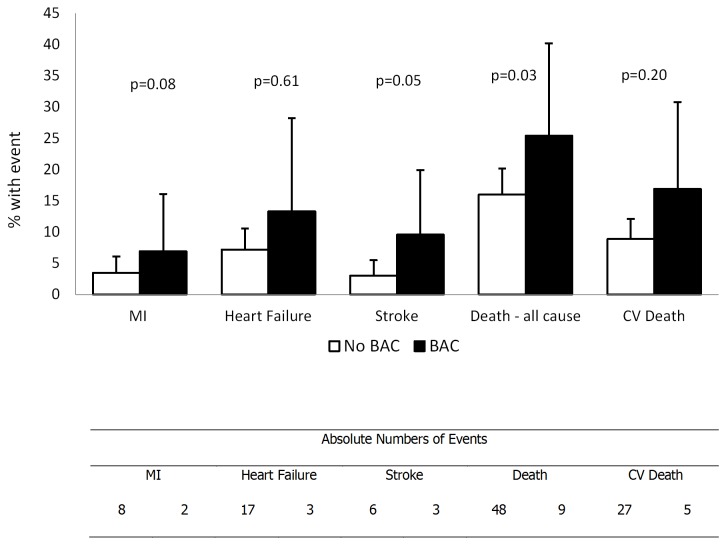
Adverse Outcomes at a Median of 6.8 years and 9.5 Years of Follow-Up.

The women with BAC had higher rates of individual and composite events than did those without BAC (Figure 2). Despite the relatively low event rates, the difference for stroke events almost reached statistical significance (p=0.056). Over a median of 9.5 years, women with BAC had an almost doubled death rate compared to those without BAC (p=0.010).

**Figure 3:**
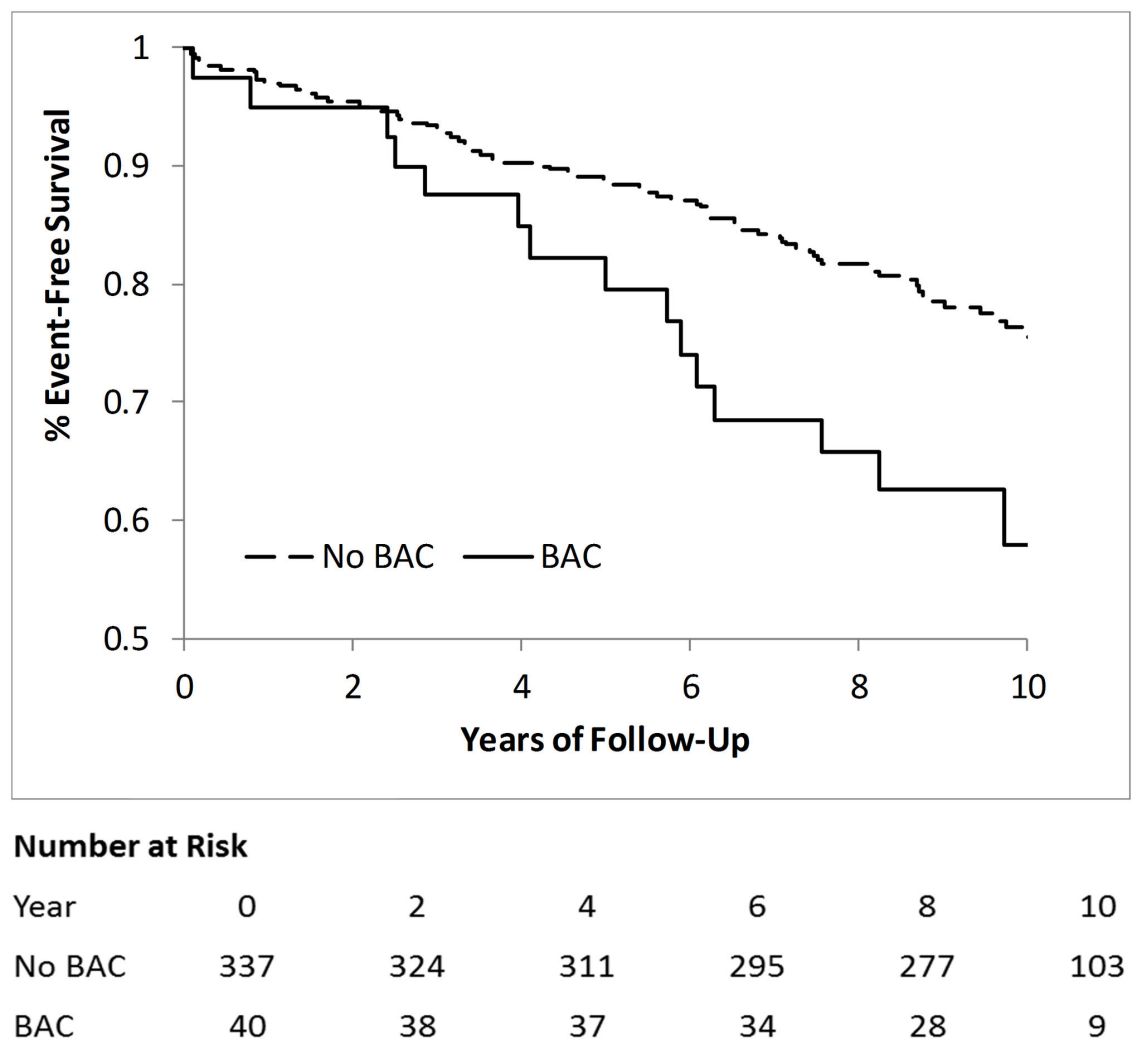
Freedom from Major Cardiovascular Events by Presence vs. Absence of BAC.

Freedom from major adverse events is shown in Figure 3. Women with BAC had a higher event rate that included all-cause mortality and non-fatal MI, heart failure, or stroke. Kaplan-Meier estimated events over 9.5 years were 22% in women without BAC and 39% in women with BAC (p=0.004).

The relationship between BAC and adverse major events remained unchanged (hazard ratio [95% confidence interval]= 2.21 [1.30, 3.78], p=0.004) in multivariate modeling controlling for the major risk factors including: age, diabetes, smoking history, and recent use of antihypertensive medications and of statins (Table 2). When entering the angiographic CAD severity score into the model, the effect of BAC was attenuated but remained statistically significant (1.96 [1.12, 3.43], p=0.018) (Table 2). In sensitivity analysis (data not shown), substituting the presence of obstructive CAD for CAD severity in the model similarly attenuated the effect of BAC but to a lesser extent (2.12 [1.24, 3.62], p=0.006).

### Response to Nitroglycerin (NTG)

We assessed brachial responses to sublingual NTG in a subgroup of women (N=334). Flow-mediated and NTG-mediated responses correlated moderately (r=0.45, p<0.0001). Further, a smaller NTG response was associated with worse outcome (HR = 0.96 (0.94, 0.98) p<.0001.

### Baseline Diameter and Events

In this cohort, baseline diameter ranged from 2.0mm to 6.4mm with a mean of 3.7mm +/- 0.7mm. The correlation between baseline diameter and FMD is -0.17 (p=0.001). Although statistically significant, this correlation is not substantively significant (in correlation analysis, the p-value is more reflective of the sample size than of the strength of association).

In women with no BAC (n=336), mean baseline diameter was 3.6mm +/- 0.7mm; whereas, in women with BAC (n=41), it was 3.9mm +/- 0.7mm (p=0.029). Therefore, collinearity between baseline diameter and BAC is likely to be minimal.

In univariate analysis, baseline diameter is predictive of major events (HR=1.05 (1.02, 1.08), p=0.0004.

On multivariate analysis, when baseline diameter is substituted for BAC in the two models in table 2, the HR drops to 1.03 (0.998, 1.06), p=0.07 in Model 1 and to 1.01 (0.98, 1.04), p=0.51 in Model 2.

Further adding BAC into the model, the p-value for baseline diameter drops to 0.15 in Model 1 and 0.73 in Model 2. At the same time, the effect of BAC is only slightly reduced: 2.07 (1.20,3.57) p=0.008 in Model 1 and 1.93 (1.10, 3.40) p=0.023 in Model 2.

## Discussion

Our results demonstrate that BAC is prevalent (11%), largely not predicted by traditional risk factors or CAD, and yet is predictive of a two-fold increased major adverse event rate, including mortality in women. In multivariate analysis, only insulin levels, pulmonary disease, and family history of CAD were independent predictors of BAC. Flow-mediated and NTG-mediated responses correlated moderately and a smaller NTG response was associated with worse outcome. Finally while baseline diameter did predict adverse events in univariate analysis, the result for baseline diameter became non-significant when added into a multivariate model while the result for BAC still remained significant.

### Prior BAC reports

FMD by BART is typically reported in prior literature as a mean value + standard deviation with no mention of the range of values. As such, it is unknown how many prior studies have encountered BAC. Several papers have reported the range of their FMD values, and document a small percentage of patients with constriction upon release of the blood pressure cuff [3,5-8]. Sondergaard et al. studied BART in 119 patients with ischemic heart disease. They report that 2 of their 31 patients with ischemic episodes and at least 6 of their 88 patients without ischemic episodes had constriction upon release of the blood pressure cuff (7% overall), many of which constricted >5% (our definition of BAC). While only 29% of their population was female, the percentage with BAC by sex was not documented [5]. Teragawa et al. studied the relationship between coronary and peripheral endothelial function (by coronary reactivity testing and BART, respectively) in 41 patients with angiographically normal coronary arteries [3]. Two of their 41 patients (5%) had constriction of the brachial artery upon release of the blood pressure cuff, however neither constricted >2%. Eighteen of their patients were women, however the sex of the 2 patients with constriction is unknown. Mitchell et al. evaluated FMD by BART in 2045 participants from the Framingham Offspring Study [6]. They too showed constriction in a small percentage of subjects (exact percentage not published), however none constricted >2%. Their data were presented by sex, and although not statistically assessed, did not suggest a dramatic difference in rates of constriction between men and women. Finally, Gori et al. have demonstrated constriction following hyperemia in several of their studies. Most notably at least 5 of 451 patients with chest pain who underwent angiography [8] and 4 of 148 patients (24 with hypertension, 24 with congestive heart failure, 24 with CAD, and 76 controls) [7] had constriction. In the latter cohort, all 4 that constricted had congestive heart failure. Again none constricted >5% and they were not differentiated by gender.

We found BAC in 11% of our patient population, all of whom were women with suspected myocardial ischemia. Since women were largely underrepresented in the above studies, it is possible that our higher rate of constriction represents a more severe vasoconstrictive response in women than men. It is also possible that our population simply represents a sicker patient population than those of Teragawa et al. and Mitchell et al. although our percentage of BAC is even higher than Sondergaard et al. who studied subjects with documented ischemic heart disease and Gori et al. who found constriction primarily amongst heart failure patients. Finally, the larger proportion of constriction in our study may be reflective of a shorter occlusion time and lower stimulus for dilation. In our study, BAC predicted a two-fold increased major adverse event rate, including mortality in women. Interestingly as seen in Figure 2, only all-cause mortality was significant as an individual endpoint. Since there was a higher prevalence of pulmonary disease in the BAC group (Table 1), it cannot be excluded that factors other than vascular reactivity could have influenced the study results.

### Proposed Mechanisms of BAC

We propose several potential mechanisms for the BAC found in 11% of our WISE population. First, since the normal response during the final 30 seconds of cuff occlusion is constriction [12], it is possible that BAC represents a failure to dilate with hyperemia as opposed to active vasoconstriction. This would suggest that BAC is a severe form of low FMD, one in which there is minimal NO-mediated dilation from severe endothelial dysfunction.

Second, it is well documented that following peak hyperemic flow, there is a time delay of FMD such that FMD occurs when flow and shear stress have returned to near resting levels. Various proposed mechanisms for this delay include a delay in NO release or an initial opposing vasoconstrictor response that is shorter lived than NO release. Jiang et al. studied these two hypotheses and supported the latter explanation by demonstrating that shear stress induced an immediate and sustained NO-dependent decrease in vascular tone that was offset by short-lived vasoconstriction [13]. This vasoconstriction was a consequence of high flow and was caused by a hydrostatic drop in mean arterial pressure along the upper limb leading to a drop in transmural pressure distending the arterial wall. While the authors concluded that FMD which is measured at 30 seconds to 2 minutes after cuff release (when flow is back to baseline) still remains a reliable measure of NO-dependent vasodilation, it is possible in our patients with BAC that this vasoconstrictor response was either stronger or more prolonged than usual outweighing any NO-mediated vasodilation.

Third, acetylcholine has been shown to evoke paradoxical vasoconstriction in atherosclerotic coronary arteries, through unopposed activation of muscarinic receptors on smooth muscle cells [1]. It is possible that BAC represents active vasoconstriction in a severely diseased vessel by a substance such as acetylcholine.

Fourth, the traditional approach to FMD calculation expresses the diameter at 60 seconds after cuff deflation relative to the preceding baseline diameter. In a study by Black et al., 42% of older subjects missed true peak dilation when FMD was assessed continuously up to 90 seconds post cuff deflation [14]. While we measured subjects up to 2 minutes post cuff deflation, it is possible that we missed peak dilation in those patients with BAC. Regardless, any constriction following cuff deflation is abnormal and even if dilation occurred farther out than 2 minutes; our current study results demonstrate that BAC at 2 minutes is associated with an adverse prognosis.

### Study Limitations

Our study has several limitations. First, due to the design of the study, causality cannot be determined or implied. Second, since clinically-indicated coronary angiography was performed in all of our study patients, our results may not be generalizable to non-angiographic populations of women. Third, our BAC subgroup size was small with few major adverse events, thus our study may not accurately estimate the true BAC-associated risk. Further, our while our mean FMD is consistent with prior studies, our standard deviation was large indicating a heterogeneous study population. Despite the resulting low statistical power, BAC emerged as a statistically significant independent predictor of major adverse events. Fourth, we did not time BART to a specific time in the menstrual cycle in premenopausal women, increasing the variability in the recordings and perhaps underestimating our results. We do not believe this has significantly influenced our results since <25% of women were premenopausal. Fifth, since a large proportion of our BART recordings either did not have Doppler or the signal was unacceptable we are unable to conclude whether the BAC we encountered truly represents flow-mediated constriction or simply a failure to dilate. Sixth, we do not have repeated scans to test the reproducibility of the BAC measurement; future work should address this.

## Conclusions

BAC was prevalent (11%), largely not predicted by traditional risk factors or CAD, and yet predictive of a two-fold increased major adverse event rate, including mortality in women. These results suggest that vasoconstriction or severe failure to dilate may play a relatively important role in ischemic heart disease pathophysiology in women. Future studies should investigate BAC in larger sample sizes, along with other measures of vasoconstriction in order to further delineate the pathophysiology and prognostic significance for women.

## References

[1] Brunner H, Cockcroft JR, Deanfield J, Donald A, Ferrannini E et al. (2005) Endothelial function and dysfunction. Part II: Association with cardiovascular risk factors and diseases. A statement by the Working Group on Endothelins and Endothelial Factors of the European Society of Hypertension. J Hypertens 23: 233-246. doi:10.1097/00004872-200502000-00001. PubMed: 15662207. 15662207 10.1097/00004872-200502000-00001

[2] Anderson TJ, Uehata A, Gerhard MD, Meredith IT, Knab S et al. (1995) Close relation of endothelial function in the human coronary and peripheral circulations. J Am Coll Cardiol 26: 1235-1241. doi:10.1016/0735-1097(95)00327-4. PubMed: 7594037. 7594037 10.1016/0735-1097(95)00327-4

[3] Teragawa H, Ueda K, Matsuda K, Kimura M, Higashi Y et al. (2005) Relationship between endothelial function in the coronary and brachial arteries. Clin Cardiol 28: 460-466. doi:10.1002/clc.4960281004. PubMed: 16274093. 16274093 10.1002/clc.4960281004 PMC6654417

[4] Holubkov R, Karas RH, Pepine CJ, Rickens CR, Reichek N et al. (2002) Large brachial artery diameter is associated with angiographic coronary artery disease in women. Am Heart J 143: 802-807. doi:10.1067/mhj.2002.121735. PubMed: 12040340. 12040340 10.1067/mhj.2002.121735

[5] Søndergaard E, Møller JE, Egstrup K (2002) Relationship between vascular dysfunction in peripheral arteries and ischemic episodes during daily life in patients with ischemic heart disease and hypercholesterolemia. Am Heart J 144: 108-114. PubMed: 12094196. 12094196 10.1067/mhj.2002.123147

[6] Mitchell GF, Parise H, Vita JA, Larson MG, Warner E et al. (2004) Local shear stress and brachial artery flow-mediated dilation: the Framingham Heart Study. Hypertension 44: 134-139. doi:10.1161/01.HYP.0000137305.77635.68. PubMed: 15249547. 15249547 10.1161/01.HYP.0000137305.77635.68

[7] Gori T, Grotti S, Dragoni S, Lisi M, Stolfo G et al. (2010) Assessment of vascular function: flow-mediated constriction complements the information of flow-mediated dilatation. Heart 96: 141-147. doi:10.1136/hrt.2009.167213. PubMed: 19858140. 19858140 10.1136/hrt.2009.167213

[8] Gori T, Muxel S, Damaske A, Radmacher MC, Fasola F et al. (2012) Endothelial function assessment: flow-mediated dilation and constriction provide different and complementary information on the presence of coronary artery disease. Eur Heart J 33: 363-371. doi:10.1093/eurheartj/ehr361. PubMed: 21920964. 21920964 10.1093/eurheartj/ehr361

[9] Merz CN, Kelsey SF, Pepine CJ, Reichek N, Reis SE et al. (1999) The Women’s Ischemia Syndrome Evaluation (WISE) study: protocol design, methodology and feasibility report. J Am Coll Cardiol 33: 1453-1461. doi:10.1016/S0735-1097(99)00082-0. PubMed: 10334408. 10334408 10.1016/s0735-1097(99)00082-0

[10] Sharaf BL, Pepine CJ, Kerensky RA, Reis SE, Reichek N et al. (2001) Detailed angiographic analysis of women with suspected ischemic chest pain (pilot phase data from the NHLBI-sponsored Women’s Ischemia Syndrome Evaluation [WISE] Study Angiographic Core Laboratory). Am J Cardiol 87: 937-941; A933 doi:10.1016/S0002-9149(01)01424-2. PubMed: 11305981. 11305981 10.1016/s0002-9149(01)01424-2

[11] Pepine CJ, Anderson RD, Sharaf BL, Reis SE, Smith KM et al. (2010) Coronary microvascular reactivity to adenosine predicts adverse outcome in women evaluated for suspected ischemia results from the National Heart, Lung and Blood Institute WISE (Women’s Ischemia Syndrome Evaluation) study. J Am Coll Cardiol 55: 2825-2832. doi:10.1016/j.jacc.2010.01.054. PubMed: 20579539. 20579539 10.1016/j.jacc.2010.01.054 PMC2898523

[12] Gori T, Dragoni S, Lisi M, Giuseppe DS, Sonnati S et al. (2008) Conduit artery constriction mediated by low flow a novel noninvasive method for the assessment of vascular function. J Am Coll Cardiol 51: 1953-1958. doi:10.1016/j.jacc.2008.01.049. PubMed: 18482663. 18482663 10.1016/j.jacc.2008.01.049

[13] Jiang B, Seddon M, Fok H, Donald A, Chowienczyk P (2011) Flow-mediated dilation of the radial artery is offset by flow-induced reduction in transmural pressure. Hypertension 57: 1145-1150. doi:10.1161/HYPERTENSIONAHA.110.163113. PubMed: 21502570. 21502570 10.1161/HYPERTENSIONAHA.110.163113

[14] Black MA, Cable NT, Thijssen DH, Green DJ (2008) Importance of measuring the time course of flow-mediated dilatation in humans. Hypertension 51: 203-210. doi:10.1161/HYPERTENSIONAHA.107.101014. PubMed: 18086954. 18086954 10.1161/HYPERTENSIONAHA.107.101014

